# Cyclability evaluation on Si based Negative Electrode in Lithium ion Battery by Graphite Phase Evolution: an *operando* X-ray diffraction study

**DOI:** 10.1038/s41598-018-38112-2

**Published:** 2019-02-04

**Authors:** Chih-Wei Hu, Jyh-Pin Chou, Shang-Chieh Hou, Alice Hu, Yu-Fan Su, Tsan-Yao Chen, Wing-Keong Liew, Yen-Fa Liao, Jow-Lay Huang, Jin-Ming Chen, Chia-Chin Chang

**Affiliations:** 1 Department of Engineering and System Science, National Tsing Hua University, Hsinchu, 30013 Taiwan; 2 Department of mechanical and biomedical engineering, City University of Hong Kong, Kowloon, Hong Kong SAR; 3 Department of Materials Science and Engineering, National Cheng Kung University, Tainan, 70101 Taiwan; 4 Department of Greenergy, National University of Tainan, Tainan, 70005 Taiwan; 5 Hierarchical Green-Energy Materials (Hi-GEM) Research Center, National Cheng Kung University, Tainan, 70101 Taiwan; 6 National Synchrotron Radiation Research Center, Hsinchu, 30076 Taiwan

## Abstract

Artificial graphite (FSN) additive is employed as internal structural label for projecting cyclability of Si material native electrode in a mass ratio of Si/FSN = 1.0 in Li ion battery (LIB). Results of *operando* X-ray diffraction analysis on Si-FSN negative electrode in LIB demonstrate that one can evaluate the lithiation and delithiation affinity of active material by referring phase transition delay of graphite as affected by experimental splits in a formation process of LIB. We prove that a thin layer of surface amorphous structure and residual lattice strain are formed in Si by high energy ball-milling treatment. Those manipulations improve Li intercalation kinetics and thus enabling a capacity fading of less than 10% (from 1860 to 1650 mAhg^−1^) for Si negative electrode in 50 cycles. Of utmost importance, this study discloses a robust assessment for revealing mechanism on amorphous and strain related silicide formation and predicting cyclability of negative electrode by quantitative phase evolution rate of FSN additive in LIB.

## Introduction

With raising demand of power consumption in transportation platforms, portable electronics, as well as high energy density modulation and storage system, high capacity materials are evitable issues for development of Li ion battery (LIB). With such a consideration, silicon based materials are no doubt the next generation negative electrode material in LIB due to its highest theoretical capacity among studied materials^1,2^. However, as compared to existing negative electrode materials, the lithium diffusion coefficient and intrinsic conductivity of Si are relatively low hindering its steps into the market^3^. Furthermore, Si possesses a volume expansion up to 400% due to a solid-state alloying reaction in lithiation (charge) process^4^. It induces extensive stress between residual Si and Li interacted domain, therefore, leading to pulverization of silicon powder in negative electrode and instability of the solid electrolyte interphase (SEI) layer^5,6^. Those characteristics pill off Si powders from current collector and increase electrical contact resistance in electrode^7–9^. Meanwhile, crashed Si powders exposes fresh surface to electrolyte thus causing overgrowth of solid electrolyte interface (ESI). In past decades, improvements in the cyclability and rate capability of silicon negative electrodes have been demonstrated by reducing particle size to the nanoscale^10^, seeking conductive coatings^11^ or conductive networks^12^, providing void space to accommodate volume expansion^13^, and introducing highly elastic phases to mitigate the deleterious effect of large volume changes^14,15^. Regardless of attempts been utilized, cyclability of LIB has been partially improved and is still far from criteria of the commercialization.

With safety and cost considerations, capacity, failure mode, and cyclability are essential inspections for commercialization of LIB. In these inspections, cyclability takes the longest time and largest resources, therefore, are long-standing issue for the slow development of LIB. Internal standard could be an efficient label as comparison for redox responses of active materials in electrochemical devices. Such a technique is conventionally employed by adding stable materials with known structure and chemical state in target sample for calibrating spatial or chemical specifications upon physical inspections (including X-ray absorption spectroscopy (XAS), X-ray diffraction (XRD), and X-ray photoemission (XPS)) and process development in semiconductor. However, internal standard is rarely being used due to the insufficient information on kinetics of structural evolutions for active materials *operando* LIB devices. In this study, artificial conducting graphite (FSN) is employed as internal structural label for verifying lithiation and delithiation affinity of active materials *operando* LIB. By cross-referencing results of density functional theory (DFT) calculation and *operando* XRD, effects of high energy milling on silicide formation and lithiation/delithiation affinity of Si materials are quantitatively revealed by changes of crystal structure of FSN as a function of applied potential of LIB. Notice that, priority of phase evolution between Si and graphite domains are dominated by their affinity to lithiation. Consequently, obtained results not only disclose the Li ion insertion/extraction behaviours in negative electrode but provide a new class of internal standard method for projecting quality and cyclability of silicon-based materials in formation stage of LIB.

## Results and Discussion

### Physical Structure Characterizations on Si based materials

**Figure 1:**
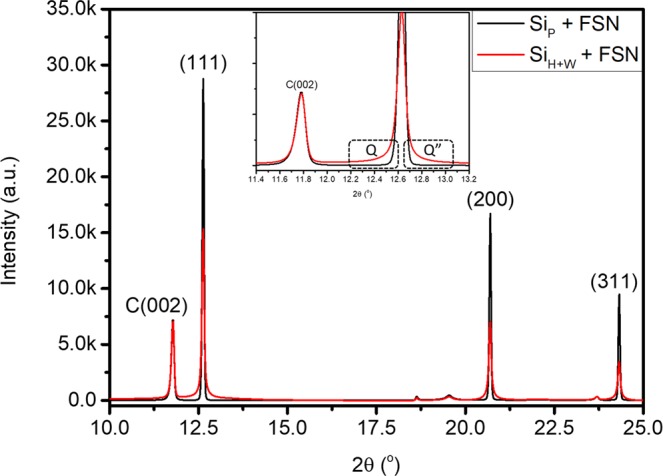
XRD patterns of as-prepared Si_P_ + FSN and Si_H+W_ + FSN electrodes. Inset presents room in for the two diffraction patterns in a range from 11.4° to 13.2°. Wavelength of incident X-ray is 0.689 Å (18 keV).

Crystal structure of Si_P_ and Si_H+W_ is revealed by using XRD analysis. Structure parameters are quantitatively determined by Rietveld refinement on their diffraction patterns (Fig. S1) and corresponding lattice parameters of Si_P_ + FSN and Si_H+W_ + FSN are listed in Table S1. Accordingly, lattice parameter is 5.435(6) Å and unit cell volume (V) is 160.598(9) Å^3^ for Si_H+W_. For Si_P_, lattice constant is 5.431(3) Å and unit cell volume is 160.218(0) Å^3^. As a result, one can notice a slight distortion and lattice expansion by 0.24% on Si crystal (Si_H+W_) by high energy ball-milling treatment. Diffraction patterns of the two electrode are compared in Fig. 1 and intensity is normalized by that of FSN (002) peak. Accordingly, all diffraction peaks of Si_H+W_ + FSN possess lower intensity, broader peak width accompanied with stronger diffusion scattering background (denoted by Q and Q” in inset) as compared to those of Si_P_ + FSN. As consistently proved by high resolution transmission electron microscopy (HRTEM) images in Fig. S2 and XRD analysis (Table 1), those characteristics can be ascribed for formation of amorphous Si species, certain short range disorder structure (sub-nanometer domains), and increased preferential (111) facets in Si_H+W_ by HEMM. Quantitative structural parameters are summarized in Table 1. Accordingly, crystal structure of Si phase remaining unchanged by HEMM treatment. For Si_P_ + FSN, average coherent length (D_avg_) is 834.9 Å for (111), 790.5 Å for (220), and 757.1 Å for (311) facets in Si phase. After HEMM treatments, D_avg_ of Si_H+W_ + FSN is respectively reduced by 20% in (111) facet (624.2 Å), ~43% in (220) facet (451.7 Å), and 46% in (311) facet (455.4 Å) as compared to those of Si_P_ + FSN. In a meantime, H_(111)_/H_(220)_ and H_(111)_/H_(311)_ is respectively increased to 2.215 and 4.493 revealing its significant preference along Si (111) facet as compared to that of ideal Si crystal and Si_P_. Changes of those parameters resembles the “damage” of Si phase by high energy treatment and thus increasing the asymmetric crystal ratio in (111) facet.

**Table 1:** XRD analysis determined structure parameters of Si_P_ + FSN and Si_H+W_ + FSN.

Sample	Facet	2θ	D_avg_ (Å)	d (Å)	H_(111)_/H_(hkl)_**
Si_P_ + FSN	(002)*	11.787	445.4	3.355	
	(111)	12.634	780.2	3.130	
	(220)	20.698	790.5	1.917	1.724
	(311)	24.326	849.1	1.635	3.031
Si_H+W_ + FSN	(002)*	11.787	445.4	3.355	
	(111)	12.634	624.2	3.130	
	(220)	20.698	451.7	1.917	2.215
	(311)	24.315	455.4	1.636	4.493

*diffraction peak of FSN (002) facet. **H_(111)_/H_(hkl)_ denotes the ratio of peak intensities for (111) and (hkl) facets. In an ideal Si crystal with a symmetric group of 227, H_(111)_/H_(220)_ is 1.497 and H_(111)_/H_(311)_ is 1.794. (source: Materials Project ID: mp-149/, 10.17188/1190959).

### Interface Properties of Experimental Si based Negative Electrode Materials by DFT calculation and EIS characterization

**Figure 2:**
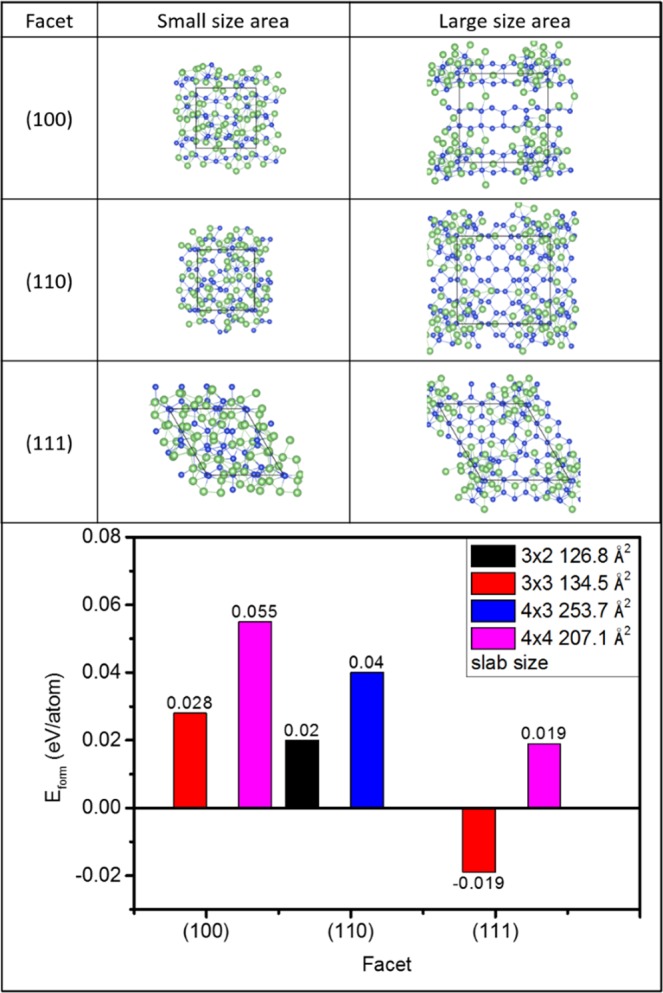
DFT calculated atomic packing structure and corresponding formation energy (E_form_ (eV/atom)) for models comprising two Li_15_Si_4_ clusters in Si slabs surface with facets indexed by the first three diffraction peaks of experimental samples.

Affinity of Li silicide formation is a crucial factor in performance of Si materials as negative electrode in LIB. This factor is a combination results including atomic packing density of facets, size of Si crystal, and anchoring ligands as dock for Li intercalation. Effects of proposed high energy milling treatment on Li silicide formation affinity are evidenced by cross-referencing DFT (with structure models determined by XRD fitting) and XRD determined crystal structure evolution of experimental materials in *operando* LIB. Figure 2 compares top view of DFT calculated atomic packing structure for models of Li_15_Si_4_ clusters in Si slabs and corresponding formation energy (E_form_ (eV/atom)) with facets indexed by the first three diffraction peaks in Fig. 1. Accordingly, the two Li_15_Si_4_ clusters built from its primitive bulk structure (ICSD no.167674) are homogeneously packing on surface with a slab size of 116.5–134.5 Å^2^. Such a result can be rationalized by steric effects when two Li_15_Si_4_ clusters are stacked in a surface with an area smaller than their close packed dimension (cross section area). Formation energies (E_form_) are −0.019 (eV/atom) for (111), 0.02 (eV/atom) for (220), and 0.028 (eV/atom) for (311) facets. A negative value of E_form_ indicates an exothermic reaction meaning the strong preference of silicide formation in (111) facet when slab size is 3 × 3 (134.5 Å^2^). As for models with slab sizes of 207.1 to 253.7 Å^2^, E_form_ follows the same trend to that of model with small slabs (126.8 to 134.5 Å^2^). Among them, E_form_ of (111) is 0.019 (eV/atom). This value is reduced respectively by 52.5% and 65.4% as compared to that of (220) (0.04, eV/atom) and (311) (0.055, eV/atom) facets again showing a strong preference of silicide formation in (111) facet regardless the presence of steric effects in Si phase interface. Preference of silicide formation is further revealed by the homogeneous distribution of atomic packing scheme in (111) facet. For rest of two facets ((220) and (311)), silicide clusters tend to accumulate in slab corners. Given that accumulation of silicide occurs both in slabs with a large (253.7 Å^2^) and small (207.1 Å^2^) areas, steric effects should be a minor factor in Li silicide formation as compared to facet selectivity (i.e., selectivity of facet) in crystal surface once improper atomic packing configuration is adopted. With proper atomic packing configuration (i.e., (111) facet), E_form_ is substantially reduced by 0.038 (eV/atom) with slab size by ~70 Å^2^ (from 207.1 to 134.5 Å^2^). Above interpretations could be explained by effects of interface lattice mismatch and atomic arrangements on heterogeneous crystal growth of silicide in a Si crystal surface. For growing polycrystalline thin films on a crystal with completely different structure symmetry, increasing surface atomic packing density reduces local steric barrier for the subsequent position of film atoms. On the other hand, with a large local steric barrier in opened facets (i.e., (220) and (311)) reduction of E_form_ with slab size is insignificant and is because of the higher energy for silicide to packing in surface defects. It is surely that presence of surface defects facilitates Li intercalation to growth Li silicide, however, such assessment and effect might lead to side effects (such as electrolyte decomposition and Si oxidation, etc.) and are not the topic to be discussed in this study.

**Figure 3:**
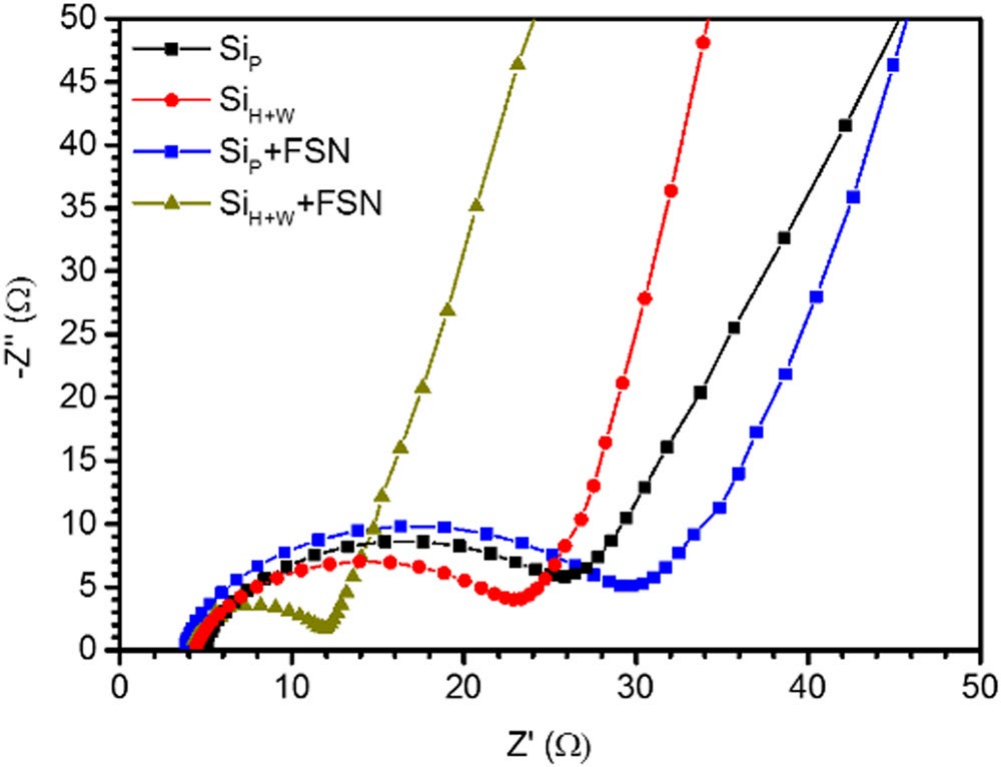
Nyquist plots for Si_P_, Si_P_ + FSN, Si_H+W_, and Si_H+W_ + FSN as negative electrode in as-prepared LIB coin cells.

Effects of preferential facet and particle size (predicted by DFT calculation and XRD analysis) on Li intercalation and Li silicide formation are complementary revealed combining electrochemical and *operando* XRD analysis on LIB with experimental Si materials (Si_P_ + FSN and Si_H+W_ + FSN) as negative electrode. Figure 3 displays the Nyquist plots of electrochemical impedance spectra (EIS) obtained from experimental Si based negative electrode at 0% lithiated state. These cells exhibited a semicircle in high frequency and a straight line in low frequency ranges, which are attributed to kinetics of charge transfer in interface and Li ions diffusion in bulk electrodes, respectively. As can be seen, a lower electrode impedance of Si_H+W_ indicates its smaller interfacial charge-transfer resistance (Rct) as compared to that of Si_P_. Such a phenomenon reveals the facilitation of Li intercalation in Si_H+W_ surface due to the surface amorphization (probed by HRTEM images), preferential (111) facets, and reduced D_avg_ (at (220) and (311) facets) in Si_H+W_. In a meantime, a uniform and local disordered amorphous Si thin layer (as consistently proved by HRTEM and XRD analyses) is formed in fresh silicon surfaces by wet milling which provides an easy access for Li intercalation. In addition, physical impacts of high energy mechanical milling and wet milling process reduce the primary and agglomerate size which shorten the Li ion diffusion length in a silicon particle. Presence of FSN provide additional Li storage sites. Those Li storage sites share the redox loading and thus reduce chemical stress in Si surface. As denoted by reducing of semicircle diameters, presence of FSN modulates the redox kinetics and thus reduces impedance of Li intercalation in silicon-based electrode.

### Internal standard structure labelling on Li affinity of Si based materials

**Figure 4:**
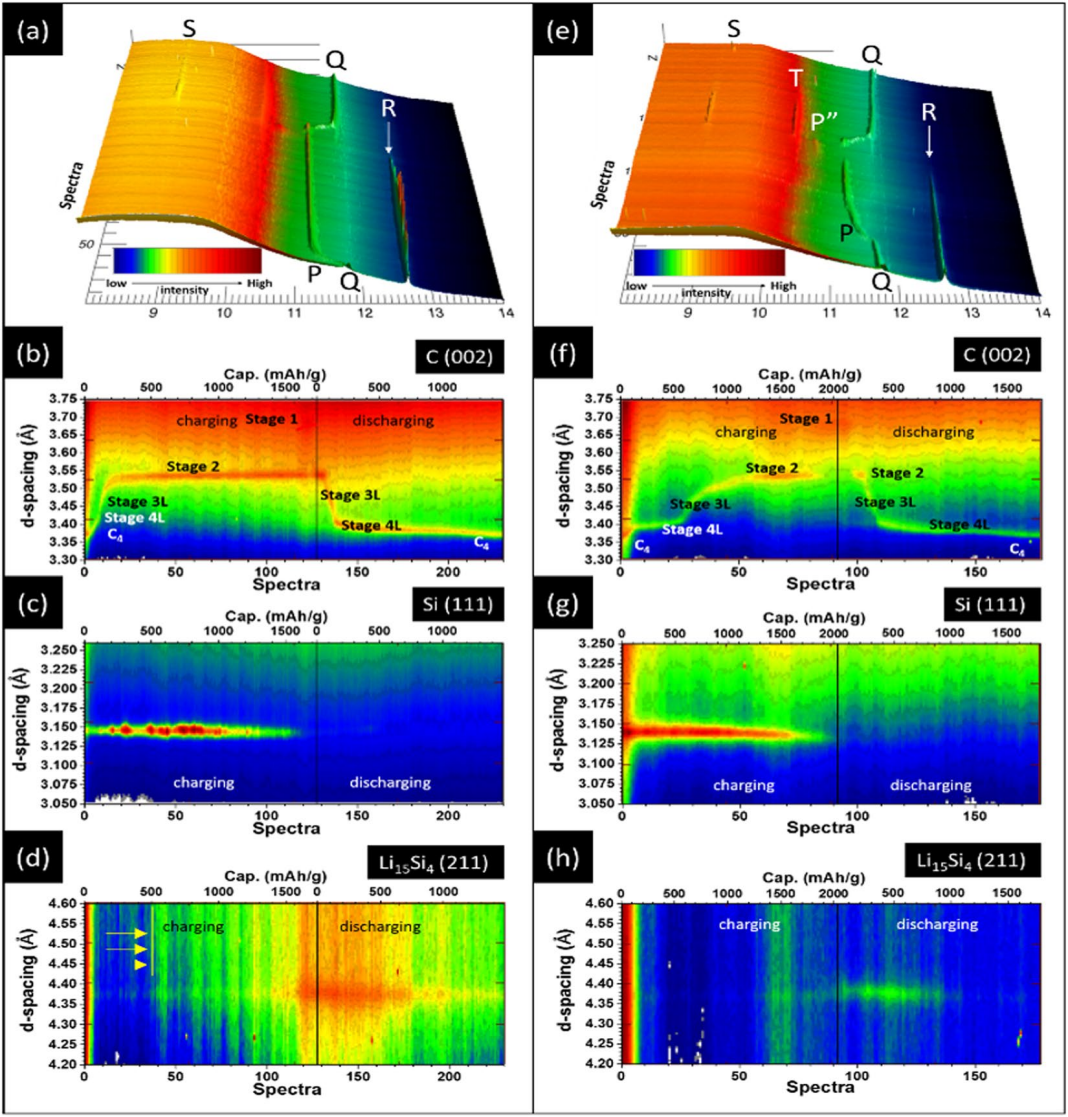
*In-situ* XRD patterns (top panel), and changes of d_(002)G_ (second row), d_(111)Si_ (third row), d_(211)Li15Si4_ (bottom row) for (**a**–**d**) Si_P_ + FSN and (**e**–**h**) Si_H+W_ + FSN as negative electrodes in LIBs in the first lithiation and delithiation processes at a rate of C/6.

Synchrotron based *operando* XRD analysis further confirms the effects of crystal size, facet selectivity on silicide formation affinity, therefore, projects cyclability of Si materials in LIB. XRD patterns of LIBs containing negative electrodes of Si_P_ + FSN and Si_H+W_ + FSN in the first lithiation/delithation cycle are compared in Fig. 4a,e, respectively. As can been seen, the three peaks at 11.3° (P), 11.8° (Q), and 12.6° (R) are diffraction lines from LiC_12_ (002), graphite (002) (C (002)), and Si (111) facets. Rest of peaks at 9° and 10.48° are diffraction lines for (211) (peak S) and (220) facets (peak T) of Li_15_Si_4_. For Si_P_ + FSN (Fig. 4a), intensities of diffraction peaks for silicon phase gradually decrease with increasing lithiation ratio to 50%. After that, Si phase is dramatically vanished and Li_15_Si_4_ crystal phase by the end of lithiation. In delithiation process, intensity of peak S and T is decreased indicating a gradual extraction of Li-ions from Li_15_Si_4_. As consistent revealed in literature, no silicon peaks are found at the end of delithiation process. This phenomenon can be rationalized by formation of amorphous Si due to a severe Li retention. As compared to those of Si_P_ + FSN, structure evolutions of Si and silicide phases go even faster in Si_H+W_ + FSN (Fig. 4e).

For clarifying lithiation affinity of Si_P_ + FSN (Si_H+W_ + FSN), quantitative results for crystal structure evolution of graphite (C), Si, and Li_15_Si_4_ phases in the first lithiation and delithiation cycle of LIB are cross-referenced in Fig. 4b–d (Fig. 4f–h). In these figures, unit of x-axis is number of diffraction patterns, that of y-axis is d-spacing. Shown in Fig. 4b and Fig. 4f, insertion of Li ions induces a graphite phase transformations in several stages including stage 4L (whose composition is not well defined), stage 3L (LiC_24_, d_(002)G_ ~3.47 Å), dilute lattice-gas disordered stage 2 (LiC_18_), stage 2 (LiC_12_, d_(002)G_ ~3.53 Å), and stage 1 (LiC_6_, d_(001)G_ ~3.70 Å) both in Si_P_ + FSN and Si_H+W_ + FSN^16^. In those stages, numerals (1, 2, 3, and 4) refer to number of empty layers between each Li-filled layer, and different stage reflects different d-spacing^16^. By cross-referencing changes of d-spacing between crystal structures of graphite and active material, an interesting correlation to the affinity of Si with adopted treatments are revealed in Fig. 4b–d.

Figure 4b shows 2D contour for changes of inter-planar spacing of graphite (002) facet (C (002)) in Si_P_ + FSN. As shown, increasing d-spacing at graphite (002) facet (d_(002)G_) from 3.36 Å to 3.52 Å indicates a successive evolution of Li-intercalated LiC_x_ to stage 2 phase with capacity from 0 to 150 mAh g^−1^ in lithiation process. By increasing capacity to 150 mAh g^−1^, d_(002)G_ is linearly expanded to that of Li enriched stage (stage 2, LiC_12_). Given that mass ratio of FSN is 50 wt% (equivalent to an ideal capacity of ~170 mAh g^−1^) in active material, transformation of graphite to stage 2 (LiC_12_) suggests that Li ions are mostly intercalate in FSN. In this event, FSN performs a substantial higher affinity for Li intercalation as compared to that of Si_P_ in active material. After capacity higher than 150 mAh g^−1^, d_(002)G_ is slightly increased by 0.01 Å (from 3.52 to 3.53 Å) by a subsequent lithiation to 1720 mAh g^−1^. For structural interpretation in details, changes of peak intensity for Si phases in Si_P_ + FSN and Si_H+W_ + FSN with lithiation ratio are compared in Figs S3a and S3b. Accordingly, with an absence of LiC_6_ phase and vibration of Si (111) peak intensity (Fig. 4c), one can notice that most of Li is intercalated in Si phase with lithiation ratios from 15 to 100% in Si_P_ + FSN with a capacity of ~1570 mAh g^−1^. With a mass ratio of 50 wt% in active materials, this value is decreased by ~18% as compared to theoretical capacity of Si (1850 mAh g^−1^) and is possibly due to a crack of Li_x_Si phases from Si surface in a Si_P_ + FSN electrode.

In a Si_H+W_ + FSN electrode (Fig. 4e), four stages including stage 4L (100–590 mAh g^−1^), 3L (590–1200 mAh g^−1^), stage 2 (1200–1890 mAh g^−1^), and stage 1 (1900–2030 mAh g^−1^) are found in graphite phase evolution by lithiation from 0 to 100% (2030 mAh g^−1^). Among them, stage 1 is fully lithiated graphite phase and can only be formed by a kinetics balance between intercalation and diffusion rates of Li ions in graphite surface in a negative electrode lithiated higher than 94%. It is important to note that active materials possess a higher than 98% of ideal capacity of graphite and Si phases in Si_H+W_ + FSN; where capacity contribution is ~170–180 mAh g^−1^ for graphite phase and ~1800–1900 mAh g^−1^ for Si phase. Compared to that of Si_P_ + FSN, graphite phase delay with lithiation ratio indicates that affinity of Li ion to surface modified Si phase is substantial higher than that of graphite phase in Si_H+W_ + FSN. Those scenarios are direct evidences rationalizing the strong lithiation preferential of Si (111), amorphous Si, and defect regions in Si_H+W_ + FSN electrode. Facile delithiation from Si_H+W_ + FSN is consistently revealed by phase transition of graphite. Shown in Fig. 4f, d_(002)G_ hold in stage 1 by delithiation from 0 to 125 mAh g^−1^ and then move to stage 2 until 235 mAh g^−1^. Further delithiation from 235 to 375 mAh g^−1^ results in a transition from stage 3L to stage 4L in graphite. In this region, this delithiation value is doubled to that can be offered by graphite meaning that Li extraction is mainly from Si phase in a Si_H+W_ + FSN electrode. On the other hand, in delithiation process of a Si_P_ + FSN electrode (Fig. 4b), stage 2 to stage 4L transition is found by delithiation from 0 to 160 mAh g^−1^ meaning that most of capacity is contributed from graphite phase (i.e., activation energy for Li extraction from graphite is lower than that from Si phase). Preference of lithiation/delithiation remaining hold even at the 50^th^ cycles and is consistently revealed by comparing changes of d_(002)G_ between Si_P_ + FSN and Si_H+W_ + FSN with respect to lithiation ratios of LIBs (Fig. S3).

**Figure 5:**
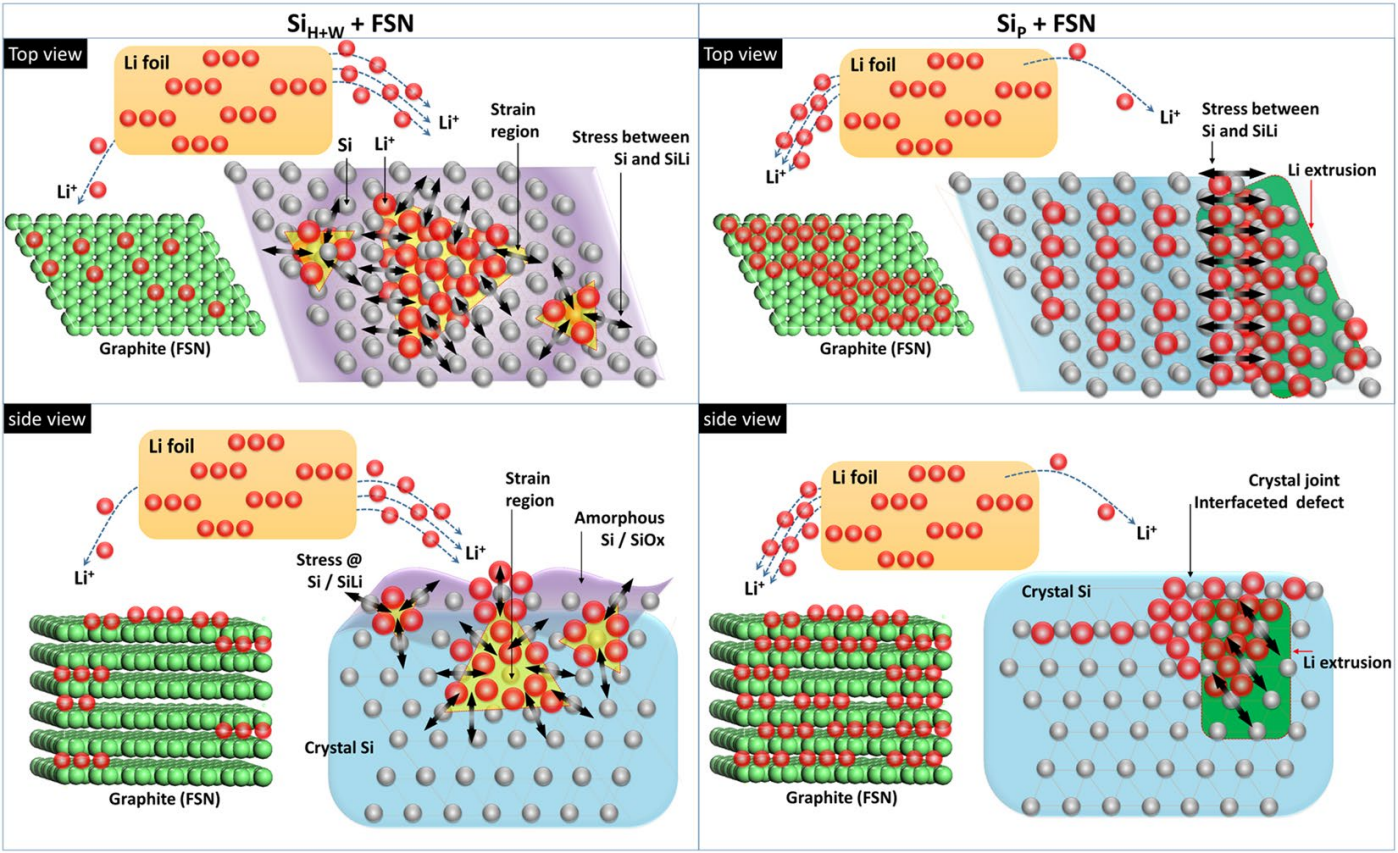
Schematic representation to Li intercalation manners in Si materials with (Si_H+W_) and without (Si_P_) high energy ball milling treatment. Strain regions in amorphous layer possess large local space for Li intercalation and flat surface (i.e., (111) facet) reduce formation energy for Li silicide in Si powder surface. The two pathways facilitate Li intercalation in Si_H+W_ reducing crack in Si crystal. In Si_P_, ratios of open facets (i.e., (220) and (311)) are higher than that in Si_H+W_. In open facets, silicide tend to cluster in corner region defect sites to form strong strain. In this event, Li^+^ ions would either extrude in bulk or induce a strong interface lattice mismatch to crack Si crystal.

Structure evolutions of Si phases provide complimentary information to the preferential lithiation of active materials in negative electrode of LIB. Shown in Fig. 4g, position of Si(111) peak for Si_P_ + FSN remaining unchanged in lithiation process. In this region, intensity of Si(111) peak is vibrating between 200 to 1100 a.u. with increasing capacity to 1490 mAh g^−1^ and then dramatically decreased to 0 by a subsequent lithiation till 1570 mAh g^−1^ (Fig. S3a). A dramatic vibration of peak intensity implies a crack of Si powder due to a strong lattice mismatch between Li silicide. This hypothesis is proved by presence of wide range scattering signals (denoted by yellow arrows) and diffraction line of Li_15_Si_4_ (211) by increasing capacity higher than 500 mAh g^−1^ in Fig. 4d. Schematic representation for silicide formation induced interface crack in Si_P_ is shown in Fig. 5. For Si_H+W_ + FSN, Li_15_Si_4_ (211) peak intensity is increased from 0 to 40 a.u. by delithiation from 0 to 500 mAh g^−1^ and then progressively decreased to 0 in a subsequent delithiation till 1200 mAh g^−1^ (Figs 4h and S3b). As compared to those of in Si_P_ + FSN, substantially weakened intensity with a broad width and delayed response of Li_15_Si_4_ (211) peak reveal a suppression of Li silicide. Such a characteristic can be attributed to formation of local disordered Si/SiO_x_ and increased ratio of (111) facets dimension with proper interface to facilitate Li intercalation and formation of amorphous Li silicide in Si_H+W_ + FSN surface (Fig. 1 and Table 1). Effects of HEMM treatments on facilitating Li accommodation in Si surface remaining hold in long-term cycling test till the 50^th^ cycle which again consistently proved the facilitations of silicide formation in (111) facets and reduced D_avg_ as predicted by DFT calculation and EIS analysis. Meanwhile, the same scenario on graphite phase evolutions delay proves the substantial improvement of Li intercalation/extraction performances of Si materials even hold after 50 cycles of LIB. Details of graphite evolutions in 50^th^ cycles are given in ESI (Fig. S2) and latter sessions. Rates of d_(002)G_ to lithiation ratio (Δd_(002)G_/ΔL) of negative electrodes are compared Fig. S4 and corresponding peak area (which can be serve as a qualitative index for extent of graphite phase transition) are compared in Table S2 for further revealing the graphite evolution as affected by affinity of Si to Li^+^ ions. Shown in Fig. S4a, the four Δd_(002)G_/ΔL peaks in (1) 3.9%, (2) 6.8%, (3) 8.1%, and (4) 94.8% of lithiation ratios correspond to the maximum rate for graphite phase transition (i.e., stage 4L, stage 3L, stage 2, and stage 1) in Si_P_ + FSN. The first three peaks incur 97.8% of area indicate that most of active sites in graphite phase are lithiated in a lithiation ratio of 8.1% (i.e., ~260 mAh g^−1^) for negative electrode. For the case of Si_H+W_ + FSN, the Δd_(002)G_/ΔL peaks at lithiation ratios of (1) 4.6%, (2) 28.6%, (3) 30.2%, (4) 36.1, and (5) 51.8% suggest the presence of five transient states in graphite phase of Si_H+W_ + FSN. In this event, as revealed by area of all Δd_(002)G_/ΔL peaks, most active sites (~96.1%) in graphite phase are lithiated in 36.1% of lithiation (~1240 mAh g^−1^) for Si_H+W_ + FSN. In a subsequent lithiation, a broad peak across 36.1 to 58.1% can be attributed to formation of LiC_6_ transient state. As compared to that of Si_P_ + FSN, transition of graphite phases is delayed by 20–25% of lithiation ratio in Si_H+W_ + FSN. Given that phase transition rate of active materials is dominated by their affinity to Li^+^ ion, such a phase delay again consistently revealed the improvement of Li affinity on Si phase in Si_H+W_ + FSN. Taking results of *operando* XRD analyses together, changes of graphite and Si phases with lithiation ratios in the first lithiation step of Si_P_ + FSN and Si_H+W_ + FSN can be respectively summarized in S4a and S4B. As compared to that of the first cycle, transition of graphite phase is further delayed by ~14 in Si_P_ + FSN and ~10% in Si_H+W_ + FSN. Such a scenario can be attributed to a reduced lithiation/delithiation barrier by LiSi_x_ formation again proving the concept of graphite phase evolution as internal structural label to Li^+^ ion affinity of active materials in LIB.

### Crystal structure affinity to formation of Li silicide in long-cycle LIB cells

Quantitative structural parameters on influences of surface modification to cyclability of silicon are determined by fitting the experimental diffraction patterns by the LAMP program. Results of graphite phase evolution (changes of d_(002)G_) with lithiation/delithiation ratios of Si_P_ + FSN in the 1^st^ and 50^th^ cycles are shown Fig. S5a. In lithiation process, Li^+^ ions move from Li metal to the negative electrodes. In Si_P_ + FSN, d_(002)G_ is significantly increased to a value of ~3.53 Å by increasing lithiation ratios from 5 to ~15%. Such a value is commonly known as a d-space of fully lithiated graphite (LiC_12_). Without significant differences of Si phase evolution in LIB, such a result suggests a lower diffusion barrier for Li^+^ ions in solid electrolyte interface (SEI) and graphite surface as compared to that in Si surface. For the case of Si_H+W_, situation goes to the opposite. As shown in Fig. S5b, d_(002)G_ is increased to that of stage 4L (3.37 Å) by increasing lithiation ratios from 5% to 30%. After that, d_(002)G_ is then dramatically increased to that of stage 3L (3.47 Å) when ratio of lithiation is 40% and then progressively increased to 3.53 Å (stage 2) till 100%. Hereafter, one can notice a delay of d_(002)G_ in Si_H+W_ + FSN as compared to that in Si_P_ + FSN in lithiation/delithiation processes. Such a delay on d_(002)G_ with lithiation extent coincide to an inverse proportional of formation energy of Li_15_Si_4_ in Si surface as consistently proved by DFT calculations.

Changes of d_(002)G_ with capacity in lithiation and delithiation processes of Si_P_ + FSN and Si_H+W_ + FSN in the 50^th^ cycle are respectively shown in Fig. S6c and S6d. Accordingly, trends of d_(002)G_ evolutions in Si_P_ + FSN in the 50^th^ cycle is differed from that in the 1^st^ cycle. Shown in Fig. S6c, changes of d_(002)G_ in Si_P_ + FSN is significantly retarded in the 50^th^ delithiation process as compared to that in the 1^st^ delithiation process. Such a delayed response on graphite phase and be explained by formation of significant amount of amorphous Si and retained Li silicide (i.e., irreversible capacity) in Si_P_ + FSN in long-cycle test. Meanwhile, as consistently explained by vibration of Si(111) peak intensity, loss of capacity reveals a crack (pulverization) of Si particle in Si_P_ + FSN in long cycle test which increases internal resistance between Si phases and back contact electrode. Changes of d_(002)G_ for Si_P_ + FSN and Si_H+W_ + FSN in delithiation process are compared in Fig. S6d. As depicted, Si_P_ + FSN and Si_H+W_ + FSN perform a similar decay trend on d_(002)G_. It means that graphite phase possesses a similar energy barrier for Li intercalation in both the two electrodes in the 50^th^ delithiation process. It is worth to note that, as compared to that of Si_H+W_ + FSN in the 1^st^ cycle, d_(002)G_ is suspended in stage IV by further increasing delithiation by 8–9%. It rationalizes the Li intercalation in Si phase is further facilitated in the 50^th^ cycle for Si_H+W_ + FSN in a LIB. Those results prove that formation of surface amorphous Si layer and increase ratio of (111) facet domain size with local distortion and surface modification by HEMM treatments improve the affinity of Li intercalation and extraction in Si surface (Fig. 5). Such a method is easy assessable therefore promising the development of Si based materials in LIB applications.

**Figure 6:**
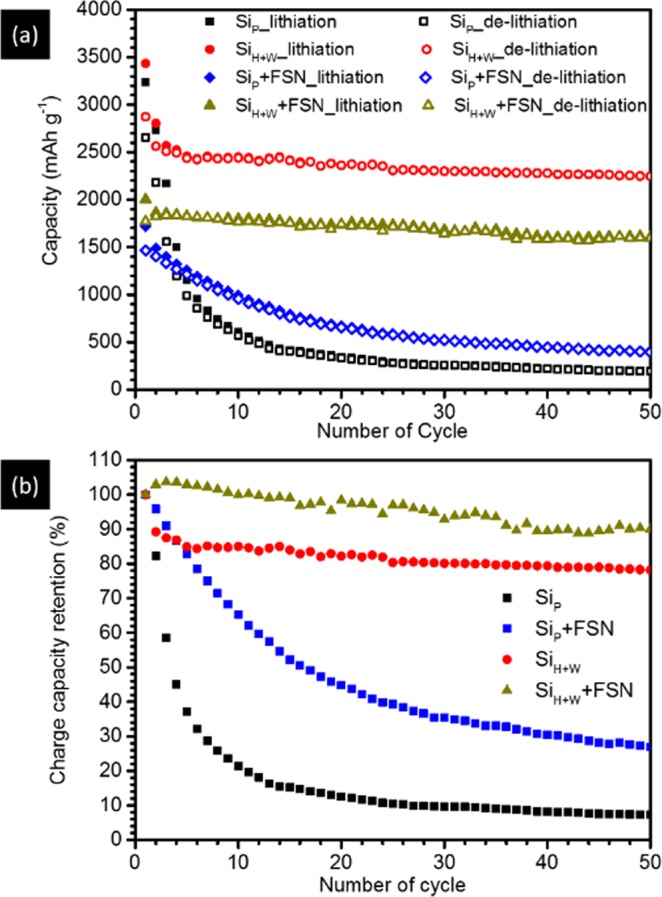
Changes of (**a**) capacity and (**b**) capacity retention with lithiation/delithiation cycles of LIBs equipped with Si_P_, Si_P_ + FSN, Si_H+W_, and Si_H+W_ + FSN negative electrodes. In these tests, rate is C/6 and potential is ranged between 0.02 V and 1.5 V vs. Li/Li^+^.

Cycle performance test further confirms the prediction on cyclability of Si materials by *operando* XRD analysis. Figure 6 shows the (a) specific capacity and (b) charging capacity retention (CR) of Si_P_, Si_P_ + FSN, Si_H+W_ and Si_H+W_ + FSN negative electrodes in LIBs till the 50^th^ lithiation/delithiation cycle. In the cycle test, rate is C/6 and potential range is 2 mV to 1.5 V. Accordingly, Si_P_ possesses a capacity of 3236 mAh g^−1^ in lithiation process and 2655 mAh g^−1^ in delithiation process in the 1^st^ cycle (formation stage). For Si_H+W_, capacity is 3470 mAh g^−1^ in lithiation and 2845 mAh g^−1^ in delithiation processes. It is important to note that both the Si_P_ and Si_H+W_ perform a columbic efficiency of ~82% in formation stage, however, with completely different fading manners of CR fading in a cycle test. For Si_P_, CR is exponentially decreased to 10% till the 50^th^ cycle. On the other hand, CR is decreases by ~15% in the first five cycles (region A) and then slightly decreased by ~4% in the subsequent cycles (region B) for Si_H+W_. As compared to that of Si_P_, a substantially reduced CR decay in region A implies a reduction of energy barrier for silicide formation therefore improving cycle stability of Si_H+W_.

Addition of conducting graphite (FSN) further enhances the difference of capacity fading mode between Si_P_ and Si_H+W_. Show in Fig. 6b, fading rate of Si_P_ is substantially reduced by mixing FSN in a weight ratio of FSN/Si_P_ = 1.0. Although capacity fading is improved by 19.6%, its CR fading remaining reaction control. On the other hand, CR fading of Si_H+W_ shows the opposite way to that of Si_P_ when mixing with FSN in the same weight ratio. In the 2^nd^ cycle, a slight increase of capacity might be attributed to the excess Li storage in FSN as compared to that of the 1^st^ cycle. Such a hypothesis is further revealed by results of *operando* XRD analysis and impedance test which proving the insufficient/uncompleted Li intercalation in FSN in the first lithiation process. Similar to that of Si_P_, CR fading of Si_H+W_ is improved by 12% by FSN additive with cycle number to 50.

## Conclusions

Graphite phase evolution is employed to evaluate cyclability of Si based materials as negative electrode in an *operando* LIB cell. At the first cycle, delay of d_(002)G_ expansion with increasing capacity reveals a strong preference of Li ion intercalation in silicon phase in negative electrode of Si_H+W_ + FSN comprising modified Si powder in lithiation process. As compared to that of Si_H+W_ + FSN, d_(002)G_ is linearly increased and stabled to that of fully lithiated graphite (3.53 Å) with respect to a capacity of ~170–180 mAh g^−1^ revealing that Li^+^ ions are mostly intercalated in graphite phase by lithiation to 5% in negative electrode of Si_P_ + FSN comprising pristine Si powder. Those scenarios are further confirmed by cross-referencing results of XRD, HRTEM, and DFT calculation indicating that performance of Si materials are improved by formation of preferential (111) facet accompanied with certain amorphous structure by HEMM treatment. A most important finding is that d_(002)G_ delay remaining hold in an *operando* LIB cell even in the 50^th^ lithiation/delithiation cycle. Such a scenario, in a fact of phase evolution dominated by Li interaction, proves that changes of d_(002)G_ with lithiation ratios is a robust qualitative index for predicting the cyclability of active materials in the formation stage of a LIB.

## Methods

### Experimental details - Sample preparation, LIB assembly, and Physical Structure Characterizations

Commercial silicon powder was purchased from Fuzhou Hokin Chemical Technology, China (Si_P_, ~10 um). To modify surface conformation of Si_P_, high energy mechanical milling (HEMM) and wet ball-milling in a planetary miller for 20 hours at room temperature were employed and resulting product is named as Si_H+W_^15^. Artificial graphite (FSN, Shanshan Technology, China, ~15 um) was employed as an internal structure standard for labelling lithiation and delithiation affinity of active materials in negative electrode of LIB. The electrochemical measurements and the investigations of structural evolution of the silicon/graphite composite electrode were carried out by using CR2032 coin-cells. Experimental negative electrodes were prepared by casting 15 μm of active materials mixture in aluminium foil. The mixture contains 65 wt% of active materials (mixture of Si_P_ and Si_H+W_ respectively with FSN at a weight ratio of silicon/graphite = 1, namely Si_P_ + FSN and Si_H+W_ + FSN), 20 wt% of carbon black (Super P), 9 wt% of poly(acrylic acid) (PAA, SigmaAldrich Co.), 3.5 wt% of styrene butadiene rubber (SBR, Zeon Co.), and 2 wt% of carboxymethylcellulose (CMC) dissolved in deionized water. Cells were assembled inside an Ar-filled glovebox. In a coin cell, counter (reference) electrode is lithium metal foil and separator is micro-porous polypropylene. The electrolyte was 1 M LiPF_6_ solution in a mixture of EC and DMC (1:1 in volume ratio). After assembly, experimental coin cells were stored for two days prior to electrochemical test. Electrochemical impedance spectroscopy (EIS) is employed to investigate the charge transfer resistance and the electronic resistance of activated material in lithium ion batteries. In this study, EIS characterization were performed on coin cells at open-circuit voltage (O_CV_) and the frequency was swept from 1 MHz to 10 mHz.

Operando XRD analysis on experimental coin cells was conducted at Taiwan beamline (BL12B2) of Spring-8 (Aioi, Japan). XRD patterns are measured with an incident x-ray wavelength of 0.688992 Å by an area detector. Pixel resolution of detector equivalents to a step size of ~0.015° in two-theta and covers a range from 0 to 30°. The system temperature is ~30 °C and exposure time is 3~4 min per pattern in transmission mode. During the data collection, the operation voltage of LIB is ranged from 2 mV to 1.5 V at a constant current (CC) of ~0.5 mA (i.e., at a rate of C/6). For conducting *operando* XRD analysis, one hole is punched and is covered with Kapton film on both top and bottom of case as X-ray window.

### Computational details

All calculations were performed in the framework of density functional theory, as implemented in the Vienna Ab initio Simulation Package^17^ code within the projected augmented wave (PAW) approach^18^. The Perdew-Burke-Ernzerhof form of the generalized gradient correction (GGA-PBE) functional is used to describe the exchange-correlation interaction^19^. The cutoff energy of 370 eV and Monkhorst-Pack k-point sampling of 12 × 12 × 12 were employed in Si bulk structure optimization, which were sufficient to obtain converged total energies. The calculated bulk lattice constant of 5.468 Å is consistent with experimental value of 5.431 Å. We utilized large slabs of Si(100)−12L, Si(110)-8L, and Si(111)-8L to simulate silicon surfaces. The bottom two layers were fixed in its bulk structure and the dangling bonds were saturated by hydrogen atoms, all the rest atoms were optimized with the convergence threshold for energy is 10–5 eV which could provide sufficient accuracy. A vacuum larger than 12 Å is used to avoid the interaction between periodic images. To clarify the affinity of Li silicide on Si surface, we performed analyses of Li_15_Si_4_ (most highly lithiated phases) on a series of Si surfaces with different size of area. The structure of Li_15_Si_4_ primitive cell consisting of thirty Li atoms and eight Si atoms is available in Inorganic Crystal Structure Database (ICSD, #167674)^20^ by utilizing database from Material Project^21^. The formation energy can be obtained from the formula: E_form_ = (E_total_ − E_surface_ − Σ*N*_i_*μ*_i_)/*N*_i_, where E_total_ and E_surface_ are the total energies of Li@Si and Si bare surface, respectively. Ni and μi are atom number and chemical potential of species i (Li and Si). Here, we used bulk energy per atom as the chemical potential.

## Supplementary information

Supplementary Information for review and publication
