# Supplementary material for: Cyclability evaluation on Si based Negative Electrode in Lithium ion Battery by Graphite Phase Evolution: an operando X-ray diffraction study
Source: Sci Rep. 2019 Feb 4;9:1299. doi: 10.1038/s41598-018-38112-2 (PMC6362206; doi:10.1038/s41598-018-38112-2)
Supplement: Supplementary file 1 — Supplementary Information for review and publication [file 41598_2018_38112_MOESM1_ESM.docx]

**Electronic Supplementary information (ESI) *for***

**Cyclability evaluation on Si based Negative Electrode in Lithium ion Battery by Graphite Phase Evolution: an operando X-ray diffraction study**

Chih-Wei Hu^a^, Jyh-Pin Chou^b^, Shang-Chieh Hou^c^, Alice Hu^b^, Yu-Fan Su^d^, Tsan-Yao Chen^a,e,*^,‡, Wing-Keong Liew^d^, Yen-Fa Liao^f^, Jow-Lay Huang^c,e^, Jin-Ming Chen^f^, Chia-Chin Chang^d,e,*,‡^

Affiliations:

1. Department of Engineering and System Science, National Tsing Hua University, Hsinchu, 30013, Taiwan. Email: chencaeser@gmail.com
2. Department of mechanical and biomedical engineering, City University of Hong Kong.
3. Department of Materials Science and Engineering, National Cheng Kung University, Tainan, 70101, Taiwan.
4. Department of Greenergy, National University of Tainan, Tainan, 70005, Taiwan. Email: juang@mail.nutn.edu.tw
5. Hierarchical Green-Energy Materials (Hi-GEM) Research Center, National Cheng Kung University, Tainan 70101, Taiwan
6. National Synchrotron Radiation Research Center, Hsinchu 30076, Taiwan

^*^To whom correspondence should be addressed:

Tsan-Yao Chen, email: [chencaeser@gmail.com](mailto:chencaeser@gmail.com) Tel: +886-3-5715131#34271;

Chia-Chin Chang, email: *juang@mail.nutn.edu.tw*

1. XRD patterns of Si – graphite composite without (Si_P_ + FSN) and with (Si_H+W_ + FSN) high energy ball milling treatment on Si powder


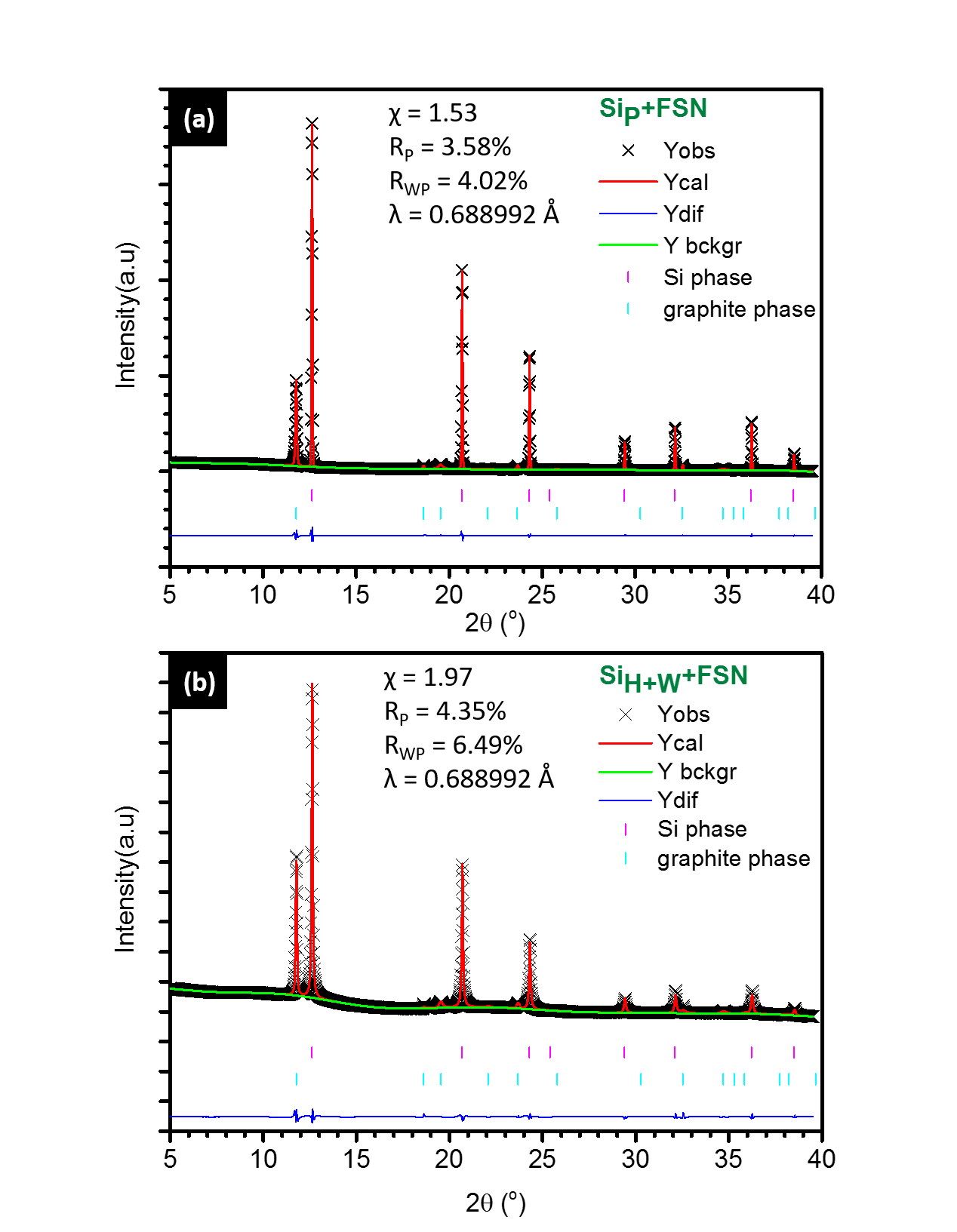


**Figure S1** Rietveld refinement of XRD patterns of (a) Si_P_+FSN and (b) Si_H+W_+FNS. The observed and calculated data are shown in black forks and red solid lines, respectively. The differences between observed data and calculated models are plotted at bottom as blue lines. The backgrounds are shown in green solid lines. Fuchsia and aqua vertical bars indicate the calculated Bragg reflections from the Silicon and graphite, respectively.

**Table S1**. Refinement lattice parameters of Si_P_+FSN and Si_H+W_+FSN.

| **Rietveld refinement results** | | | | |
| --- | --- | --- | --- | --- |
| **Sample** | Si_P_+FSN | | Si_H+W_+FSN | |
|  | Si_P_ | FSN | Si_H+W_ | FSN |
| Space group | Fd-3m | P63/mmc | Fd-3m | P63/mmc |
| **Lattice Constant (Å)** | | | | |
| a (Å) | 5.431(3) | 2.460(9) | 5.435(6) | 2.459(8) |
| b (Å) | 5.431(3) | 2.460(9) | 5.435(6) | 2.459(8) |
| c (Å) | 5.431(3) | 6.722(5) | 5.435(6) | 6.718(3) |
| alpha | 90 | 90 | 90 | 90 |
| beta | 90 | 90 | 90 | 90 |
| gamma | 90 | 120 | 90 | 120 |
| Lattice volume (Å^3^) | 160.218 | 35.259 | 160.599 | 35.204 |
| **Reliability factors** | | | | |
| R_wp_ | 4.02 | | 6.49 | |
| R_p_ | 3.58 | | 4.35 | |
| χ^2^ | 1.53 | | 1.97 | |

1. HRTEM inspection on structure of Si powders as affected by HEMM treatment

**Figure S2** compares TEM of (a) Si_W_ and (b) Si_H_ powders; where high resolution images with corresponding Fourier transformation pattern for selected area is presented in A (C), B (D), E (G), and F (H), respectively. The powders of Si_W_ and Si_H_ possess similar crystal structure (phase and coherent length) of primary particles to that of Si_P_ and Si_H+W_, therefore, are employed as benchmark sample to discuss impacts of HEMM treatment to structure evolution of Si materials in this study. For Si_W_ (**Figure S2a**), most clusters are flat or rod like structure with a length of 350 – 400 nm and a width of 80 – 120 nm comprising several primary Si_W_ nanoparticles (80 to 100 nm). Fourier transformation (FT) patterns possess identical features and denote the FCC symmetry of Si crystal both in bulk (**Figure S2a** **(C)**) and near surface region. For Si_H_ (**Figure S2b**), primary particles are agglomerated into clusters in a size around 0.5 – 0.7 μm. Within the cluster, size of crystalline phase is around 40 – 50 nm and amorphous region (denoted by yellow arrow in **Figure S2b (F)** and smeared ring in FT pattern **Figure S2b (H)**) consistently proves XRD results. Meanwhile, presence of two symmetrically aligned spot in FT pattern (**Figure S2b (C)**) suggests the preferential (111) facet in Si_H_.


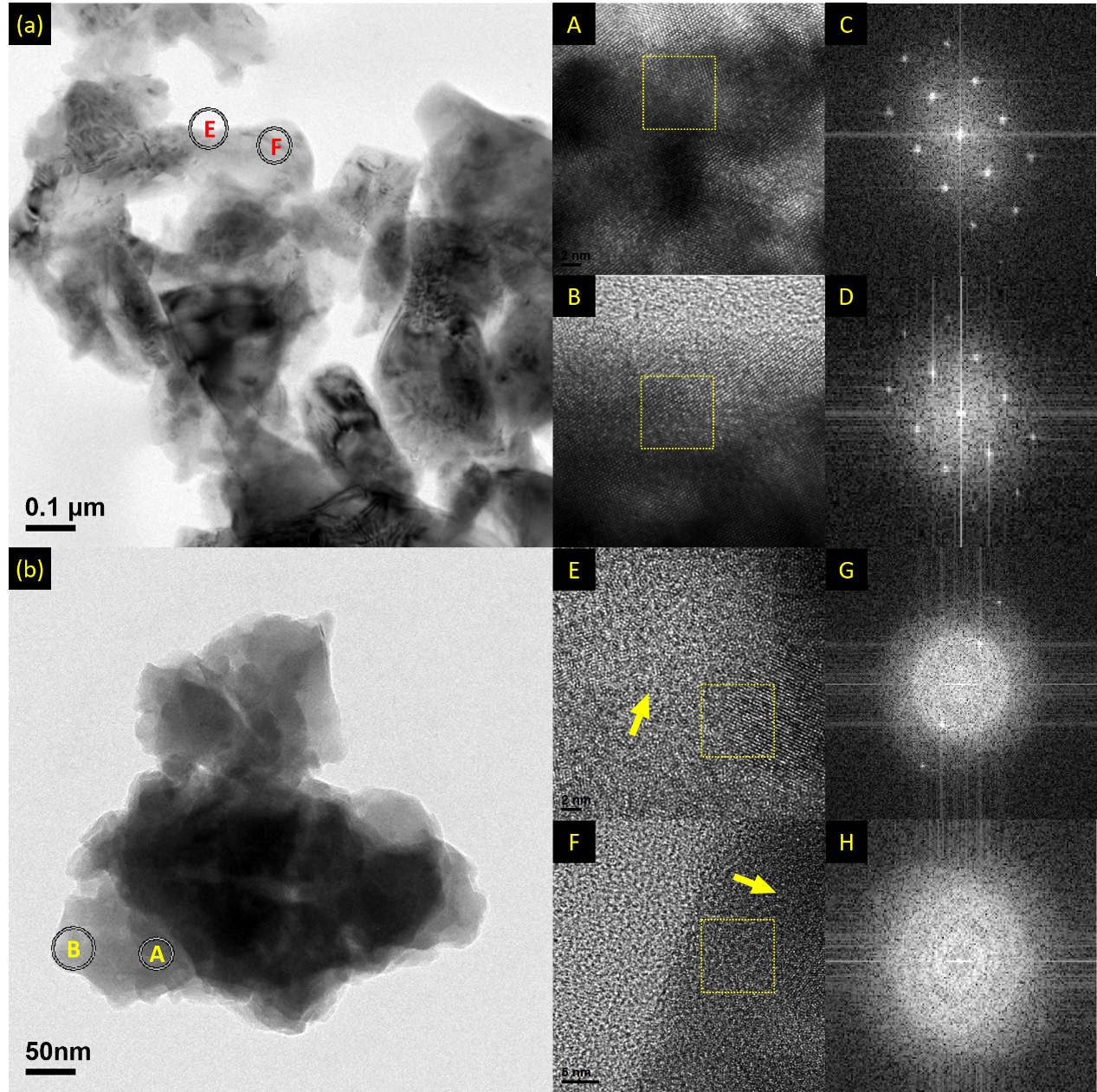


**Figure S2** (a) TEM image for Si_W_ powders with its HRTEM images in bulk (A) and near surface (B) regions and corresponding FT patterns of (C) and (D). (b) TEM image for Si_H_ powders with HRTEM images in bulk (E) and near surface (F) regions and corresponding FT patterns of (G) and (H).

1. *Operando* XRD analysis on negative electrode materials in the 1^st^ and 50^th^ cycle of LIB test


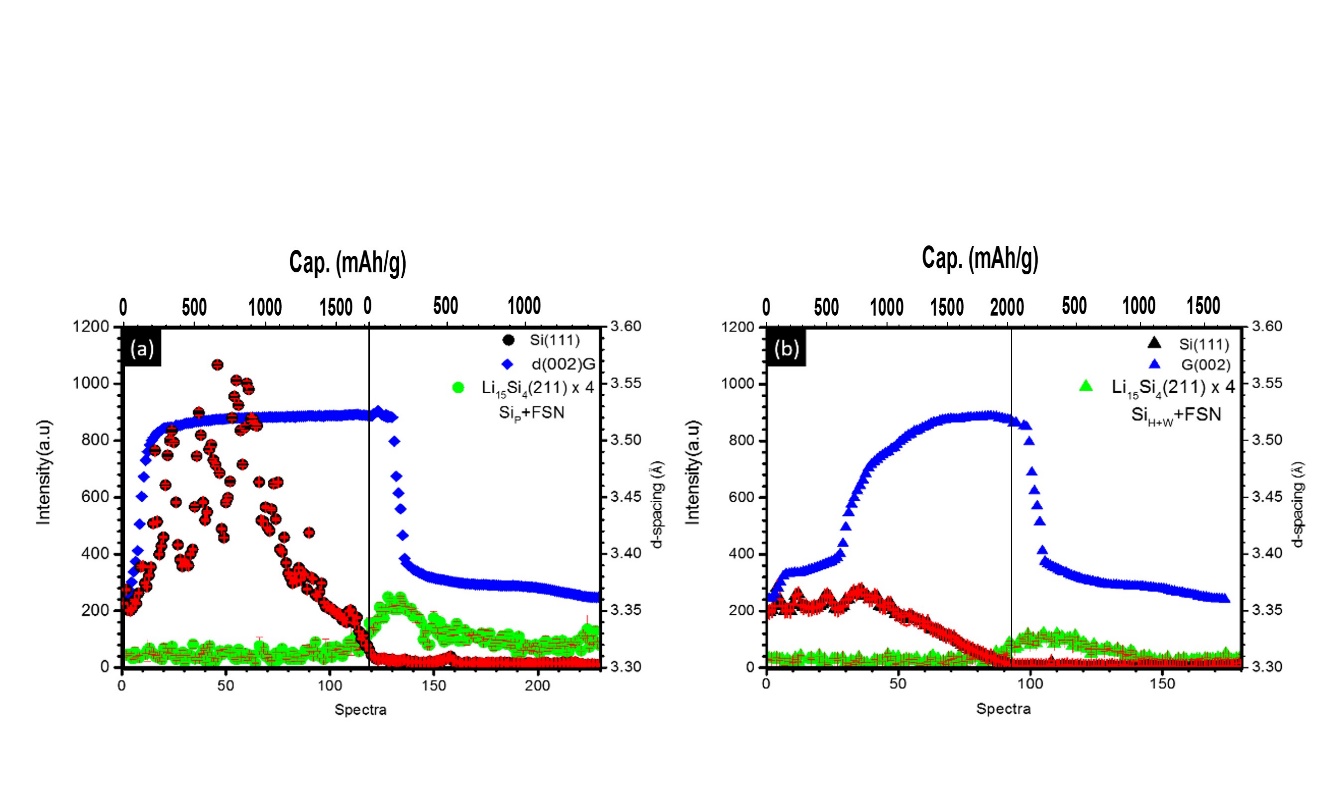


**Figure S3**. Intensity evolution of diffraction peaks in operando XRD patterns for LIB equipped with negative electrodes of (a) Si_P_+FSN and (b) Si_H+W_+FSN in the first lithiation / delithation cycle.


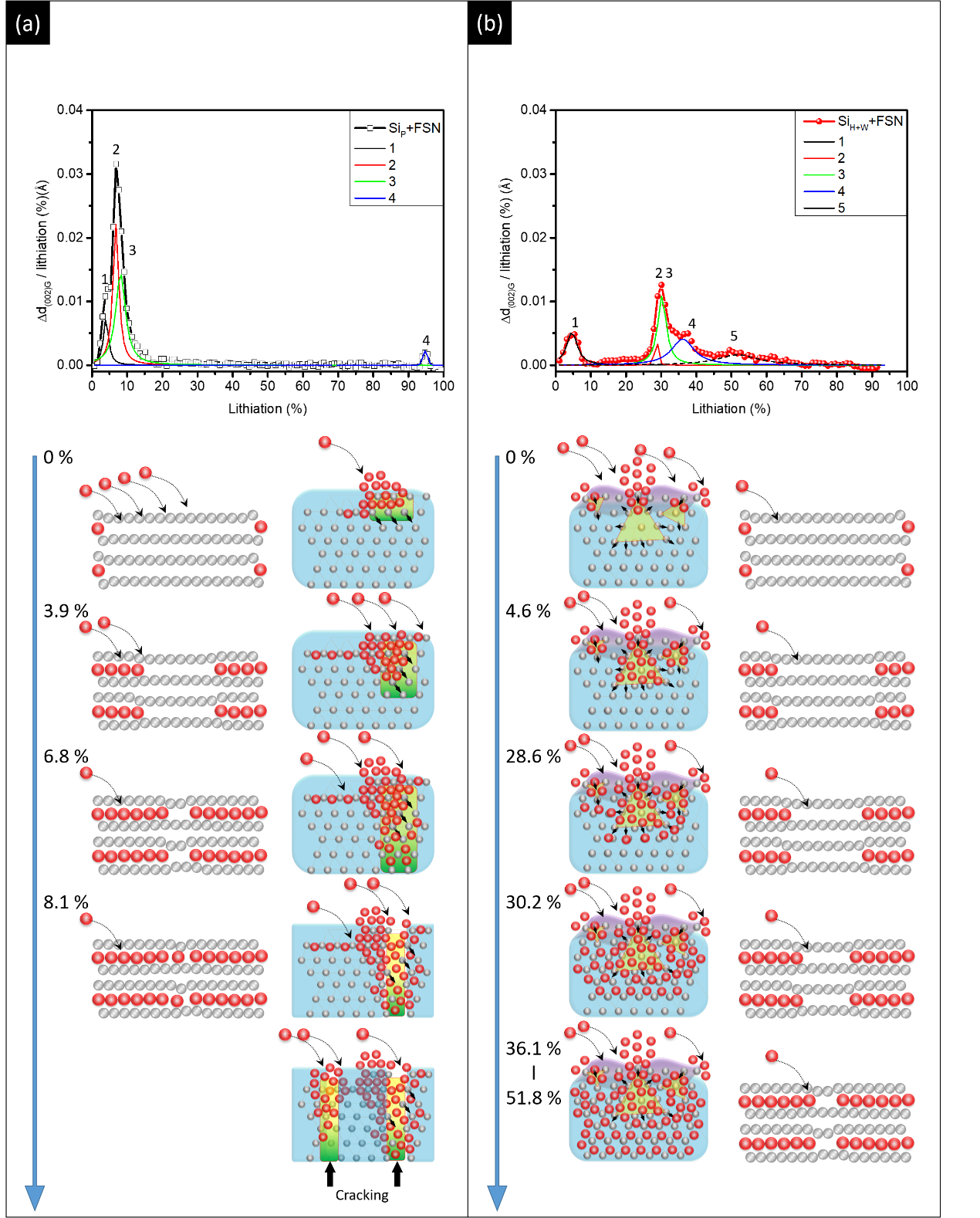


**Figure S4**. Graphite phase transition rate with lithiation ratios of (a) Si_P_+FSN and (b) Si_H+W_+FSN as negative electrode in the first lithiation / delithiation cycle of a LIB coin cell. Corresponding phase evolutions between graphite and Si phases are presented.

**Table S2** Changes of graphite phase ratio in Si_H+W_+FSN and Si_P_+FSN as a negative electrode with lithiation ratios in a LIB

| Lithiation ratio (%) | Graphite phase ratio (%) | |
| --- | --- | --- |
|  | Si_H+W_+FSN | Si_P_+FSN |
| 3.96 | NA | 11.8 (1) |
| 4.64 | 12.5 (1) | NA |
| 6.79 | NA | 41.0 (2) |
| 8.12 |  | 45.0 (3) |
| 28.61 | 47.6 (2) | NA |
| 30.26 | 26.1 (3) |  |
| 36.15 | 9.9 (4) |  |
| 51.81 | 3.9 (5) |  |
| 94.77 | NA | 2.2 (4) |

* numbers in () represent the index of transient stages of graphite phase


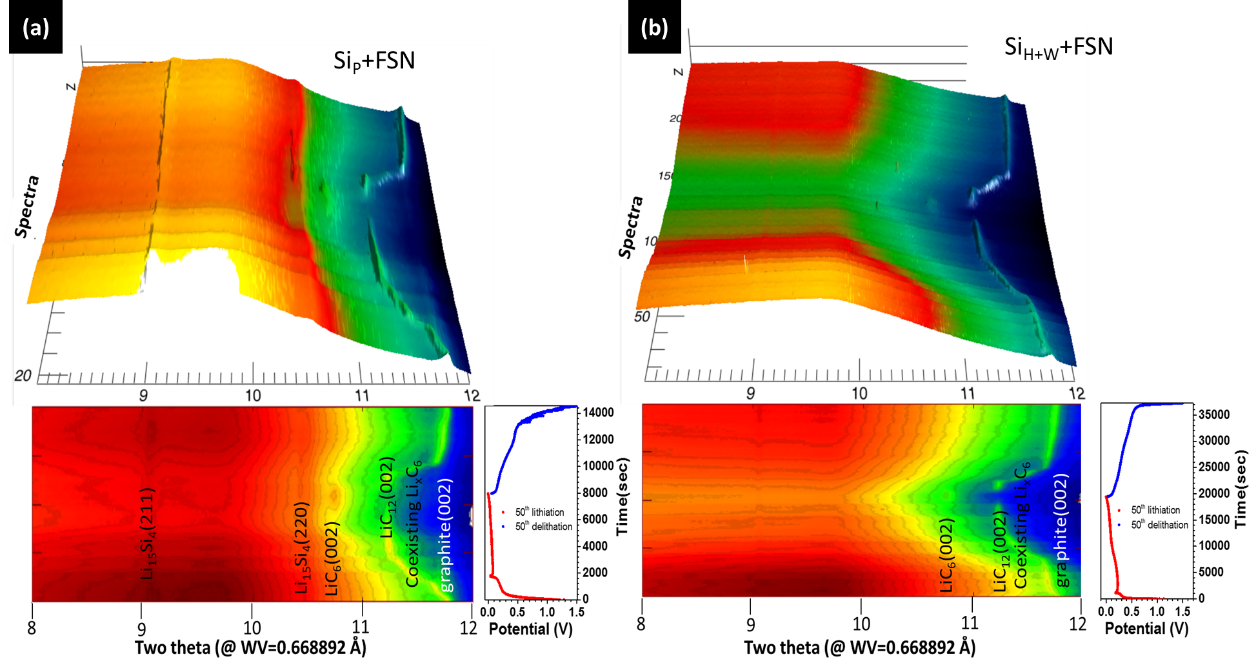


**Figure S5**. *In*-*operando* XRD patterns for (a) Si_P_+FSN and (b) Si_H+W_+FSN collected in the 50^th^ lithiation/delithiation cycle.

In **Figure S5**, all the diffraction peaks in the XRD pattern shows that the phase evolution of Si_P_+FSN and Si_H+W_+FSN undergoing the 50^th^ cycle. The peak intensity of Li_15_Si_4_(211) Bragg reflections was remained for Si_P_+FSN during lithiation and delithation process, which means the irreversibly crystalline Li_15_Si_4_ was observed due to the peeling of electrode. It is caused by large volume change and the associated lithium diffusion-induced stress during electrochemical cycling. On the other hand, the structure change of graphite (002) Bragg peak for Si_P_+FSN and Si_H+W_+FSN still have significantly different upon the 50^th^ cycle.


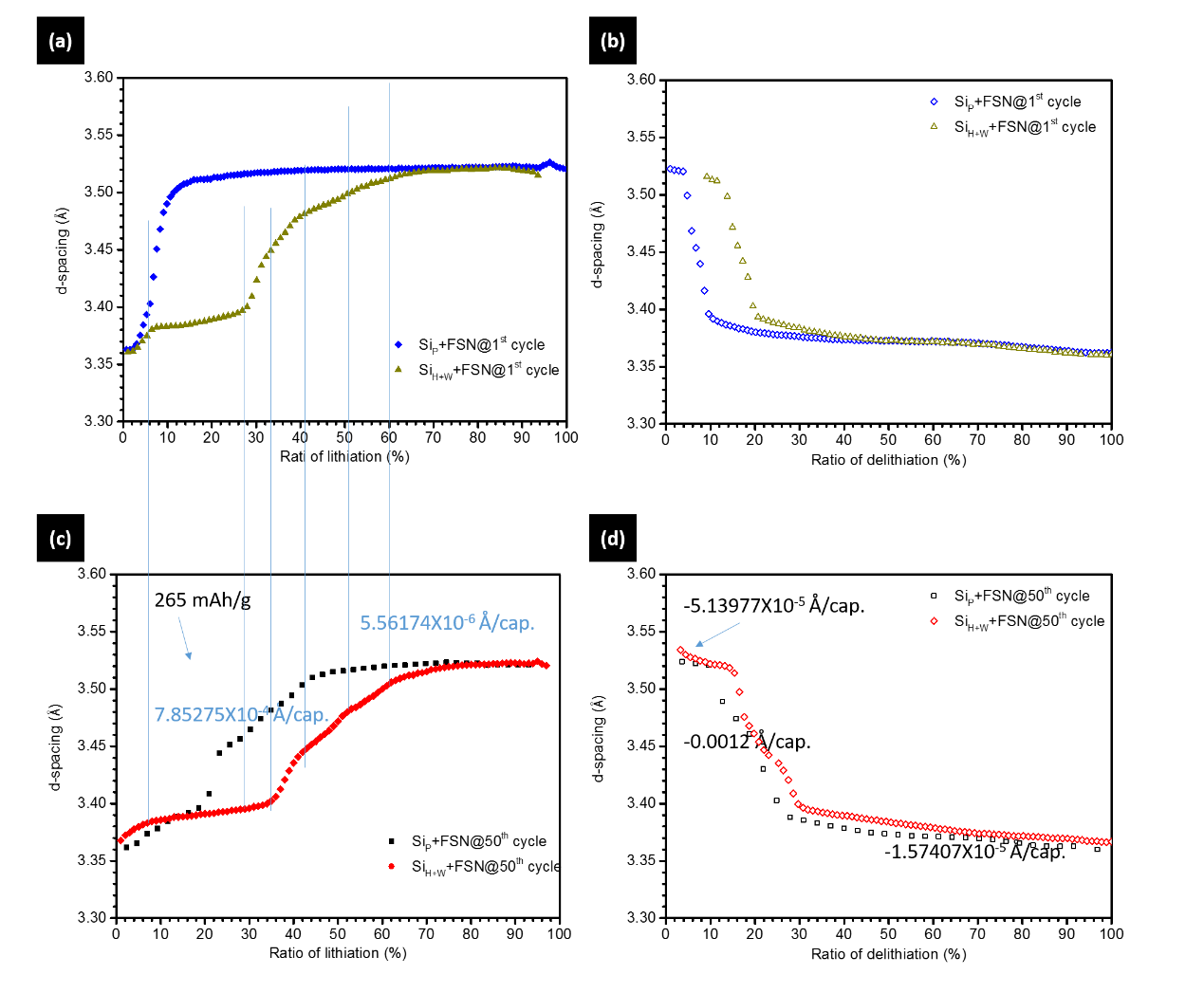


**Figure S6**. Changes of d_(200)G_ in Si_P_+FSN and Si_H+W_+FSN as a function of (**S6a**) lithiation state and (**S6b**) delithiation state in the first cycle. Corresponding plots in the 50^th^ cycle are shown in (**S6c**) and (**S6d**).


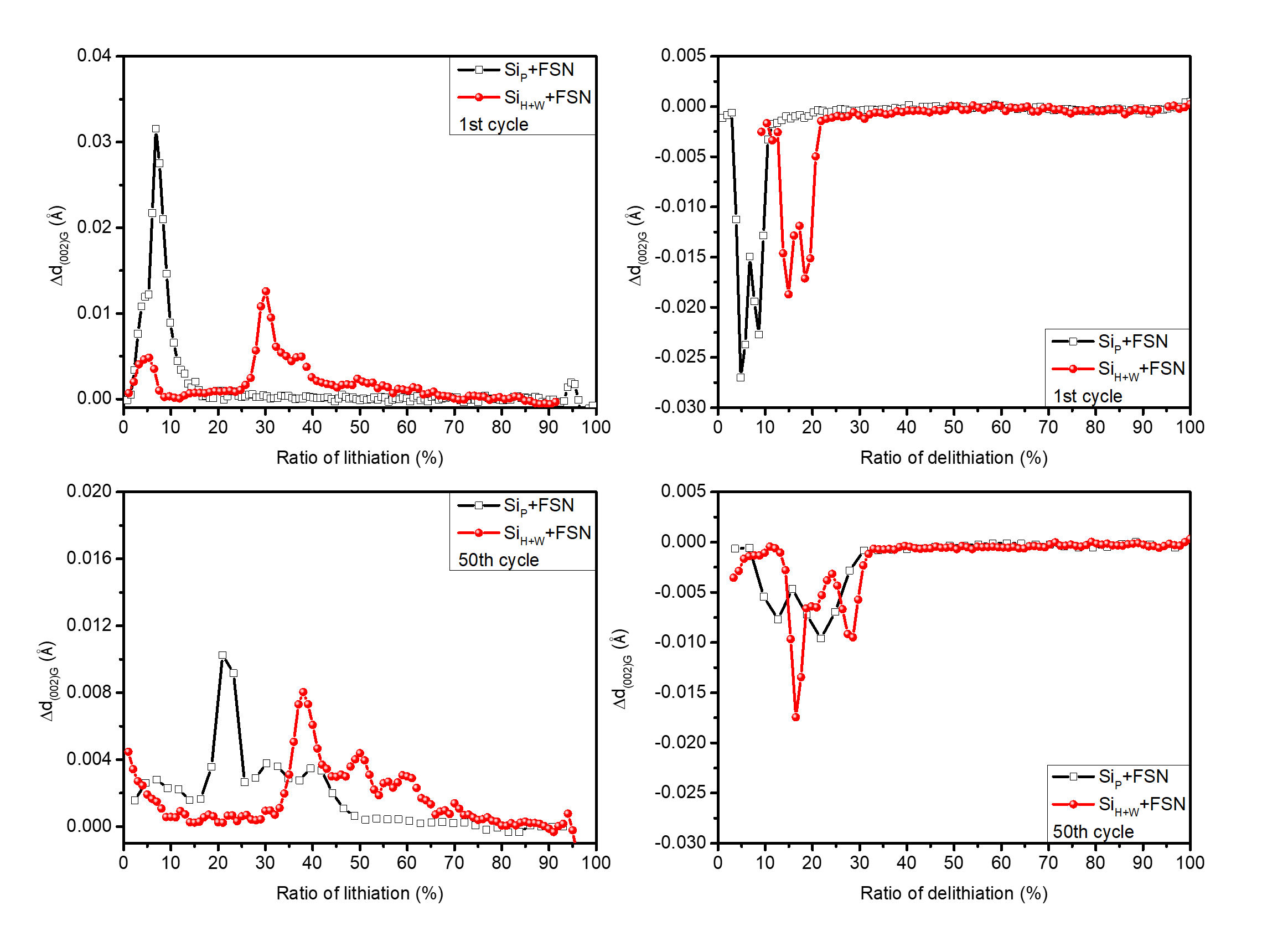


**Figure S7** Graphite phase transition rate with lithiation ratios of (a) Si_P_+FSN and (b) Si_H+W_+FSN as negative electrode in the 50th lithiation / delithiation cycle of a LIB coin cell.
